# Multifunctional Skin Dermal Extracellular Matrix Enabling Skin-Relevant Bioactivity for Tissue Remodeling, Hydration, and Anti-Hyperpigmentation

**DOI:** 10.4014/jmb.2601.01001

**Published:** 2026-01-13

**Authors:** Yu Heun Kim, Sewon Park, Jung Ho Cho, Seung Yeop Han, Seung-Woo Cho

**Affiliations:** 1 Department of Biomaterials Science and Engineering, Yonsei University, Seoul 03722, Republic of Korea; 2 Department of Biotechnology, Yonsei University, Seoul 03722, Republic of Korea; 3 Cellartgen Inc., Seoul 03722, Republic of Korea

**Keywords:** Decellularized skin-derived extracellular matrix, Anti-skin aging, ECM remodeling, hydration, Anti-melanogenic effect

## Abstract

Dermal extracellular matrix (ECM) deterioration is a central driver of skin aging, contributing to impaired elasticity, decreased moisturization, and uneven pigmentation. However, commonly used single-component ingredients and cell-derived bioactives provide limited coordinated cues and may therefore be insufficient to address these multifactorial processes. Here, we propose decellularized skin-derived ECM (skin ECM) as a multifunctional cosmetic ingredient through comparison with various existing cosmetic ingredients. Proteomic analysis shows that skin ECM retains diverse collagen subtypes along with glycoproteins and proteoglycans associated with dermal tensile properties and matrix regulation, more closely reflecting native dermal matrisome diversity than commercial collagen products. Skin ECM at an optimal concentration most effectively upregulates the expression of genes involved in ECM remodeling and hyaluronan-mediated hydration in human dermal fibroblasts. In comparative supplementation assay, skin ECM enhances fibroblast metabolic activity and induces the strongest expression of key ECM- and hydration-related genes among all tested ingredients. Interestingly, skin ECM reduces the expression of melanogenesis-related markers and melanin accumulation in melanoma cells under experimental conditions with α-melanocyte stimulating hormone treatment. Collectively, these findings highlight the potential of skin ECM for cosmetic applications to improve overall skin conditioning and its broader promise for anti-skin aging.

## Introduction

Skin aging is largely driven by progressive deterioration of the dermal extracellular matrix (ECM). Intrinsic aging and extrinsic factors such as ultraviolet (UV) exposure, disrupt dermal homeostasis, resulting in reduced collagen content, disorganized collagen fibrils, elastic fiber fragmentation, and decreased hyaluronan-mediated water retention [1-5]. These dermal structural and biochemical alterations manifest clinically as wrinkles, loss of elasticity and firmness, dryness and dysregulated melanogenesis, leading to uneven skin tone and hyperpigmentation [1, 6, 7]. Accordingly, current cosmetic development increasingly aims to focus on reinforcing dermal matrix integrity, improving hydration and pigmentation control.

Many conventional skin-relevant bioactive ingredients, including single growth factors, antioxidant vitamin derivatives, retinoids, nucleic acid-based materials, extracellular vesicles, and collagen supplements, have been explored for skin conditioning and anti-aging applications [8-15]. Although these approaches can modulate and enhance specific biological activities, they often offer limited range of coordinated cues and may therefore be insufficient to support multifactorial regulation required for overall dermal homeostasis.

Decellularized ECM has emerged as a compelling strategy for achieving tissue-mimetic functionality [16, 17]. By removing cellular constituents, decellularization can reduce cell-derived variability and immunogenicity while effectively preserving tissue specific biochemical and mechanical cues [18, 19]. Previous studies have shown that decellularized skin-derived ECM (skin ECM) preserves both skin-specific structural and compositional features and provides instructive bioactive signals that regulate dermal cell adhesion, migration, survival and matrix remodeling [20, 21]. In addition, skin ECM applied as a soluble treatment in human dermal fibroblasts has been reported to have the potential for supporting cutaneous regeneration by promoting fibroblast migration and facilitating wound healing process [22]. In this context, skin ECM is expected to better sustain intricate dermal ECM-associated processes than single component materials or cell-derived bioactives, thereby enabling more skin-like cellular responses.

In this study, we demonstrated that skin ECM more closely reflects compositional complexity of human skin than commercially available collagen products based on proteomic profiling. We further confirmed the cytocompatibility of skin ECM and identified an optimal concentration that maximally enhances hydration- and remodeling-associated cellular activity. In comparative supplementation assays, skin ECM at the optimal dose upregulated key hydration- and matrix-remodeling related genes relative to other comparator components. Moreover, skin ECM suppressed α-melanocyte stimulating hormone (MSH)-induced melanogenic gene expression and reduced intracellular melanin accumulation compared with other single-component ingredients and cell-derived materials. In summary, these findings support skin ECM as a biomimetic and multifunctional cosmetic ingredient with greater potential than conventional single-component bioactive materials and cell-derived bioactive ingredients to improve overall skin condition by enhancing skin moisturization and firmness/elasticity while also providing brightening benefits.

## Materials and Methods

### Protein Sample Preparation and Liquid Chromatography with Tandem Mass Spectrometry (LC-MS/MS) Analysis

Decellularized skin ECM (Regenix Skin, #SKdE-1G; Cellartgen, Republic of Korea), recombinant human collagen III (rhCollagen; Bloomage Biotech, China) and type I collagen-enriched VitroCol (Advanced BioMatrix, USA) were prepared for proteomic analysis through a filter-aided sample preparation (FASP) digestion kit (Abcam, UK). Proteins were reduced in 50 mM ammonium bicarbonate containing 10 mM DL-dithiothreitol (DTT; Sigma-Aldrich, USA) at 60°C for 30 min. Alkylation was subsequently performed with 55 mM iodoacetamide (IAA) in a urea solution for 30 min at room temperature with light exclusion. Proteins were then digested with MS-grade trypsin (Promega, USA) at 37°C for an overnight incubation. Resulting peptides were purified using Pierce peptide desalting spin columns (Thermo Fisher Scientific, USA), concentrated to dryness for 6 h using a centrifugal evaporator (CVE-3000, Eyela, Japan) and resuspended in 0.1% (v/v) formic acid (TCI, Japan) with 2% (v/v) acetonitrile (Thermo Fisher Scientific). Peptide amounts were determined by a Pierce quantitative colorimetric peptide assay (Thermo Fisher Scientific), and 2 μg of peptides were subjected to LC-MS/MS per injection. LC-MS/MS was performed using Orbitrap Exploris 240 coupled to Dionex U 3000 RSLCnano HPLC system (Thermo Fisher Scientific). In this process, 0.1% (v/v) formic acid in 2% (v/v) acetonitrile was used as solvent A, and 0.1% (v/v) formic acid in acetonitrile was used as solvent B. Samples were initially loaded onto Acclaim PepMap 100 trap column (100 μm × 2 cm, nanoViper C18, 5 μm, 100 Å, Thermo Fisher Scientific) and washed with solvent A for 6 min at a flow rate of 4 μl/min. The separation was subsequently performed on PepMap RSLC C18 column (75 μm × 15 cm, nanoViper C18, 3 μm, 100 Å, Thermo Fisher Scientific), with a flow rate of 300 nl/min. The gradient was applied as follows: 2% to 8% solvent B (10 min), 8% to 30% solvent B (55 min), 90% solvent B (4 min), and 2% solvent B (20 min). Full scans were recorded in the Orbitrap analyzer over m/z range of 350–1800 with a resolution of 60000 at m/z 200.

### Proteomic Identification and Quantification

MS raw files were analyzed with MaxQuant (v2.7.0.0; Max Planck Institute of Biochemistry, Germany) [23]. Database searches were carried out against UniProt proteomes (release 2025.10), using *Sus scrofa* entries for skin ECM and *Homo sapiens* entries for rhCollagen and VitroCol. Trypsin was defined as the digestion enzyme. Carbamidomethyl was set as a fixed modification, while N-terminal acetylation and methionine oxidation were included as variable modifications. Peptide and protein identifications were filtered at a false discovery rate of 1% (FDR = 0.01). Label-free quantification (LFQ) was performed with match-between-runs. Protein abundance was further represented using intensity-based absolute quantification (iBAQ) method. To compare relative composition among samples, relative iBAQ (riBAQ) values were computed by dividing each protein iBAQ by the total iBAQ value within the corresponding sample.

For comparison with native skin tissue, a quantitative proteome dataset of human suprapubic skin tissue was used to assess overall proteome and matrisome composition [22, 24]. Matrisome assignment was based on the MIT Matrisome Project database [25], and “skin-expressed-proteins” were defined using the Human Protein Atlas [26]. Gene ontology enrichment was conducted via the PANTHER overrepresentation test, and only terms with FDR < 0.05 were considered statistically significant. Protein-protein interaction (PPI) networks were obtained from STRING and visualized in Cytoscape [27, 28].

### Cell Culture

Human neonatal dermal fibroblasts (Thermo Fisher Scientific) at passages below 7 were maintained in high glucose Dulbecco’s Modified Eagle Medium (DMEM; Thermo Fisher Scientific) supplemented with 10% (v/v) fetal bovine serum (FBS, Thermo Fisher Scientific) and 1% (v/v) penicillin-streptomycin (P/S) at 37°C in a humidified incubator with 5% CO_2_. For supplement treatments, cells were maintained in low serum medium containing 2% FBS to better isolate the effects of tested supplements. The B16F10 murine melanoma cell line was grown in high glucose DMEM supplemented with 10% (v/v) FBS and 1% (v/v) P/S at 37°C in a humidified incubator with 5% CO_2_.

### Medium Supplement Test

Solubilized skin tissue ECM was prepared as a 5 mg/ml pre-gel solution by pepsin-mediated enzymatic digestion of the lyophilized ECM, followed by sequentially mixing with triple distilled water (TDW) and 10x phosphate-buffered saline (PBS; Sigma-Aldrich) on ice. The resulting solution was then neutralized to physiological pH by adding 0.5 M sodium hydroxide (NaOH; Sigma-Aldrich). For medium supplement, the solubilized skin ECM was gently pipetted to ensure the homogenous distribution and diluted in culture medium to a final concentration of 25 μg/ml prior to cell treatment. Fibroblast growth factor (FGF)-mimetic peptide (ActivePep FG100; International Nomenclature of Cosmetic Ingredients (INCI): butylene glycol & acetyl sh-tripeptide-3 amide; Herbnoori, Republic of Korea) and epidermal growth factor (EGF)-mimetic peptide (ActivePep EF100; INCI: butylene glycol & acetyl sh-tripeptide-3 amide; Herbnoori) were administered at final concentration of 2 μg/ml. A stock solution of retinyl palmitate (RP; INCI: vitamin A palmitate; Whatsoap, Republic of Korea) was prepared in 100% ethanol and stored at -20°C until use. RP was diluted in culture medium to a final concentration of 10 μg/ml. Polydeoxyribonucleotide (PDRN; Herbnoori), rhCollagen, VitroCol, ethyl ascorbic acid (EAA; INCI: ethyl ascorbyl ether; Herbnoori), and vitamin E acetate (VEA; INCI: tocopheryl acetate; Herbnoori) were applied at final concentration of 25 μg/ml. For stock solution preparation, rhCollagen was freshly prepared in DMEM immediately prior to use, whereas EAA and VEA were prepared in TDW and 100% ethanol, respectively, and stored at -20°C until use. Human dermal fibroblast-derived exosome (HDF exosome; STEMON, Republic of Korea) and *Centella asiatica* callus-derived extracellular vesicle (CAcEV; INCI: *C. asiatica* callus extracellular vesicles; GFC Life Science, Republic of Korea) were treated at 1 × 10^5^ particles/ml. A stock solution of HDF exosome was prepared in filtered 1× PBS and stored at -20°C until use. Nicotinamide mononucleotide (NMN; INCI: nicotinamide mononucleotide; Biospectrum, Republic of Korea) was treated at a final concentration of 1.6 mM, and its stock solution was prepared in filtered 1× PBS and stored at -20°C until use. For the preparation of adipose-derived stem cell conditioned medium (ADSC-CM), human ADSCs (StemPro Human Adipose-Derived Stem Cells; Invitrogen, USA) were initially cultured in T175 flasks with MesenPRO RS basal medium (Invitrogen) supplemented with MesenPRO RS growth supplement (Invitrogen) and 1% (v/v) GlutaMAX supplement (Thermo Fisher Scientific). At cell confluency of 50-60%, the culture medium was replenished with basal medium. The ADSC-cultured medium was then collected at cell confluency of 80-90%. To prepare the ADSC-conditioned medium, the collected ADSC-cultured medium was serially centrifuged at 300 g and 3,000 g for 30 min each at 4°C and filtered through a 0.2 μm pore size syringe filter (Corning, USA) to remove cell debris. The medium was stored at 4°C and used as a soluble medium supplement at 5% (v/v) for subsequent experiments. All supplements were administered on day 1 and day 3 post seeding and fibroblasts were analyzed on day 4 post seeding.

### Live/Dead Assay

Fibroblasts treated with skin ECM at 1-25 μg/ml were stained with a LIVE/DEAD Viability/Cytotoxicity Kit (Thermo Fisher Scientific). Fluorescence images were acquired with a microscope (IX73, Olympus, Japan), and live and dead cells were counted using Image J software (National Institutes of Health, USA). Viability was expressed as the percentage of live cells relative to the total cell number.

### Cell Metabolic Activity Assay

Fibroblasts were seeded in 96-well plates at 3000 cells per well. Cells were treated twice with low serum (2% FBS) medium containing the indicated supplements at 24 and 72 h post-seeding. After 96 h, cell viability was assessed using Cell Counting Kit-8 (Abcam). Water-soluble tetrazolium-8 (WST-8) reagent was added and incubated for 2 h, and absorbance was measured at 450 nm using a microplate reader (Tecan, Switzerland).

### Quantitative Real-Time Polymerase Chain Reaction (qPCR)

Total RNA was extracted from fibroblasts and melanoma cells using the TaKaRa MiniBest Universal RNA Extraction kit (TaKaRa, Japan) and complementary cDNA was synthesized using cDNA synthesis kit (TaKaRa). qPCR was then performed with SYBR Green Fast qPCR Mix (ABclonal, USA) on a StepOnePlus Real-time PCR system (Thermo Fisher Scientific). The following primers (all obtained from Bioneer, Republic of Korea) were used for qPCR to compare gene expression level: human elastin (*ELN*) (Forward: 5’-GGTATCCCATCAAGGCCCC-3’, Reverse: 5’-TTTCCCTGTGGTGTAGGGCA-3’), human hyaluronan synthase 1 (*HAS1*) (Forward: 5’-TACTTTTGGGGATGACCGGC-3’, Reverse: 5’-AAGTACGACTTGGACCAGCG-3’), human hyaluronan synthase 2 (*HAS2*) (Forward: 5’-TGACAGGCATCTCACGAACC-3’, Reverse: 5’-GGGTCTGCTGGTTTAGCCAT-3’), human fibronectin (*FN1*) (Forward: 5’-ACCTACGGATGACTCGTGCTTTGA-3’, Reverse: 5’-CAAAGC CTAAGCACTGGCACAACA-3’), mouse tyrosinase (*Tyr*) (Forward: 5’-ATCGGCCAACGATCCCATTT-3’, Reverse: 5’-TAGGTGCATTGG CTTCTGGG-3’), mouse tyrosinase-related protein-1 (*Tyrp1*) (Forward: 5’-AAGGTTACAGTGCTCCCACG-3’, Reverse: 5’-GGTTTGTCCTCC CGTTCCAT-3’), and mouse tyrosinase-related protein-2 (*Tyrp2*) (Forward: 5’-TCCTGAATGGGACCAATGCC-3’, Reverse: 5’-CAGGCATCT GTGGAAGGGTT-3’). Target gene expression was normalized to human glyceraldehyde 3-phosphate dehydrogenase (*GAPDH*) (Forward: 5’-TCCAAAATCAAGTGGGGCGA-3’, Reverse: 5’-AAATGAGCCCCAGCCTTCTC-3’) and mouse *Gapdh* (Forward: 5’-GGAGAGTGTTTCCTC GTCCC-3’, Reverse: 5’-ATCGGCCAACGATCCCATTT-3’) using the comparative C_T_ (ΔΔC_T_) method.

### Melanin Content Assay

B16F10 cells were cultured in 12-well plate for 24 h. After removing the medium, cells were pretreated for 1 h with high glucose, phenol red-free DMEM medium (Thermo Fisher Scientific) supplemented with 10% (v/v) FBS and 1% (v/v) P/S, containing indicated comparator supplements or skin ECM. Subsequently, 100 nM α-melanocyte-stimulating hormone (α-MSH; Sigma-Aldrich) was added, and cells were cultured for additional 72 h. For intracellular melanin quantification, the cultured cells were harvested and centrifuged at 4000 rpm for 5 min. Collected cells were lysed in 1 M NaOH containing 10% dimethyl sulfoxide (DMSO; Wako, Japan) and absorbance was measured at 405 nm using a microplate reader.

### Statistical Analysis

Data are reported as mean ± standard deviation (SD), as specified in figure legends. All statistical analyses were performed in GraphPad Prism 10 (GraphPad Software, USA). Differences among groups were evaluated using one-way analysis of variance (ANOVA) with Tukey’s multiple comparisons test. *n* denotes the number of biological replicates, as indicated in the figure legends.

## Results

### Skin ECM Retains Skin-Mimetic ECM Complexity for Multifunctional Skin Conditioning

**Fig. 1:**
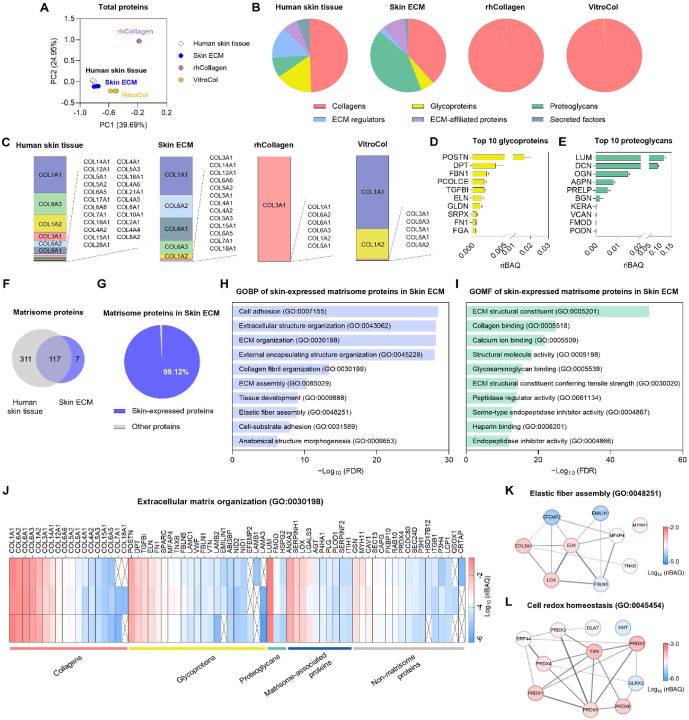
Proteomic analysis of skin ECM reveals skin-mimetic ECM components and functional properties required for the skin microenvironments. (**A**) PCA based on the total protein profiles of human skin tissue, skin ECM, rhCollagen, and VitroCol. (**B**) Pie charts depicting the relative proportions of matrisome subcategories in human skin tissue, skin ECM, rhCollagen, and VitroCol. (**C**) Relative abundance of collagen subtypes in human skin tissue, skin ECM, rhCollagen, and VitroCol. Top 10 most abundant (**D**) glycoproteins and (**E**) proteoglycans in skin ECM. (**F**) Venn diagrams showing the number of shared and unique proteins between human skin tissue and skin ECM. (**G**) Proportion of skin-expressed proteins among matrisome proteins identified in skin ECM (approximately 99.12% of the total matrisome protein content). Skin-expressed proteins were defined by the Human Protein Atlas. Top 10 most significantly enriched gene ontology terms for (**H**) biological process and (**I**) molecular function based on the skin-expressed matrisome protein set shown in (**G**). (**J**) Heatmap showing the expression of proteins associated with extracellular matrix organization (GO:0030198) in skin ECM (*n* = 3). Proteins are categorized into collagens, glycoproteins, proteoglycans, matrisome-associated proteins (ECM regulators, ECM-affiliated proteins, and secreted factors), and non-matrisome proteins. The heatmap is divided to reflect these classifications, with proteins within each category arranged in descending order of abundance. PPI networks of proteins associated with (**K**) elastic fiber assembly (GO:0048251) and (**L**) cell redox homeostasis (GO:0045454) in skin ECM. Node color indicates relative protein abundance.

Proteomic analysis using mass spectrometry was performed to compare the protein composition and comprehensive proteomic profiles of skin ECM with those of native human skin tissue and commercially available collagen products, including rhCollagen and VitroCol [24]. Principal component analysis (PCA) revealed that skin ECM was more similar to human skin tissue than rhCollagen and VitroCol in overall protein expression patterns (Fig. 1A). We next quantified the relative abundance of matrisome proteins across the six major categories. Both rhCollagen and VitroCol predominantly consisted of collagens, whereas skin ECM exhibited a more diverse distribution across multiple matrisome categories, more closely resembling the compositional profile of native dermal tissue (data from our previous study) [22] (Fig. 1B). Consistent with product specifications, rhCollagen was enriched in collagen type III alpha 1 chain (COL3A1), while VitroCol was primarily composed of collagen type I alpha 1 chain (COL1A1) and collagen type I alpha 2 chain (COL1A2) (Fig. 1C). In contrast, skin ECM contained a wider spectrum of collagen subtypes, including COL1A1, COL1A2, and multiple type VI collagens, demonstrating a closer resemblance to native human skin tissue in terms of its diverse collagen composition (Fig. 1C). Notably, distinct from the commercial collagen products, both skin ECM and native dermal tissue were enriched in type VI collagens, which play critical roles in dermal matrix assembly, fibroblast migration and behavior, and skin mechanical integrity [29, 30]. Given that skin aging is accompanied by a progressive reduction in collagen content and impaired matrix organization, the diverse collagen types retained in skin ECM may offer potential benefits for anti-aging applications [31, 32].

Beyond collagens, skin ECM also preserved glycoproteins and proteoglycans that are essential for the dermal microenvironment. Among top 10 glycoproteins, periostin (POSTN) [33], a matricellular protein that regulates ECM remodeling, procollagen C-proteinase enhancer-1 (PCPE-1) [34], which promotes procollagen processing and collagen maturation, and fibronectin (*FN1*) [35], a key mediator of dermal wound repair, are mainly associated with skin remodeling and regeneration. In addition, dermatopontin (DPT) [36], which supports collagen fibrillogenesis, and fibrillin-1 (FBN1) [37], an essential component of elastic microfibrils, help maintain dermal tissue integrity and elasticity (Fig. 1D). In particular, transforming growth factor-beta1 (TGFB1) [38-40], a multifunctional growth factor implicated in inhibition of melanogenesis and modulation of hyaluronan synthesis, was also abundantly detected in skin ECM. Furthermore, key dermal proteoglycans, including lumican (LUM) [41], decorin (DCN) [42, 43], osteoglycin (OGN) [44] and asporin (ASPN) [45], which are well known contributors to collagen fibrillogenesis and dermal tensile properties, as well as versican (VCAN) [46], a hyaluronan binding proteoglycan that supports tissue viscoelasticity, were well preserved in skin ECM (Fig. 1E). Together, these results suggest that skin ECM preserves versatile dermal glycoproteins and proteoglycans supporting matrix organization and biological functionality relevant to overall dermal homeostasis.

Moreover, of the 124 matrisome proteins detected in skin ECM, 117 were also identified in the native human skin (Fig. 1F). Consistently, 99.12% of the total matrisome proteins in skin ECM corresponded to proteins present in native human skin, supporting that skin ECM retains sufficient skin-resident matrisome components capable of providing a skin-mimetic extracellular microenvironment (Fig. 1G).

Gene ontology biological process (GOBP) analysis indicated that skin-expressed matrisome proteins in skin ECM were significantly enriched for ECM structural assembly, including collagen fibril organization and elastic fiber formation, as well as cell-matrix adhesion (Fig. 1H). Likewise, gene ontology molecular function (GOMF) analysis revealed enrichment of ECM structural and binding activities, including terms associated with tensile strength and matrix-binding functions (Fig. 1I). Collectively, these enrichments suggest that skin ECM retains functional properties relevant to dermal matrix integrity and biomechanics, supporting its potential utility as a cosmetic ingredient for improving skin elasticity and firmness. This was further supported by heatmap analysis of proteins annotated to the GOBP term “extracellular matrix organization”, a key process underlying skin elasticity, which revealed a diverse set of matrisome and non-matrisome proteins in skin ECM (Fig. 1J). In addition, PPI analysis of proteins associated with “elastic fiber assembly” revealed a cohesive interaction module with prominent connectivity among matrix-stabilizing and elastic fiber-related proteins, including *ELN*, lysyl oxidase (LOX), and fibulin-5 (FBLN5) (Fig. 1K). Finally, PPI analysis of “cell redox homeostasis” proteins identified a tightly connected thioredoxin (TXN)-peroxiredoxin (PRDX) network, with links to additional redox-associated factors, suggesting that skin ECM also retains protein networks relevant to oxidative stress regulation (Fig. 1L). Overall, these findings demonstrate that skin ECM preserves coordinated protein interactions that support ECM organization, biomechanical integrity and redox homeostasis, reinforcing its skin-mimetic potential for cosmetic anti-aging applications.

### Skin ECM Exhibits Cytocompatibility and Promotes Dermal Matrix Homeostasis at an Optimal Concentration

**Fig. 2:**
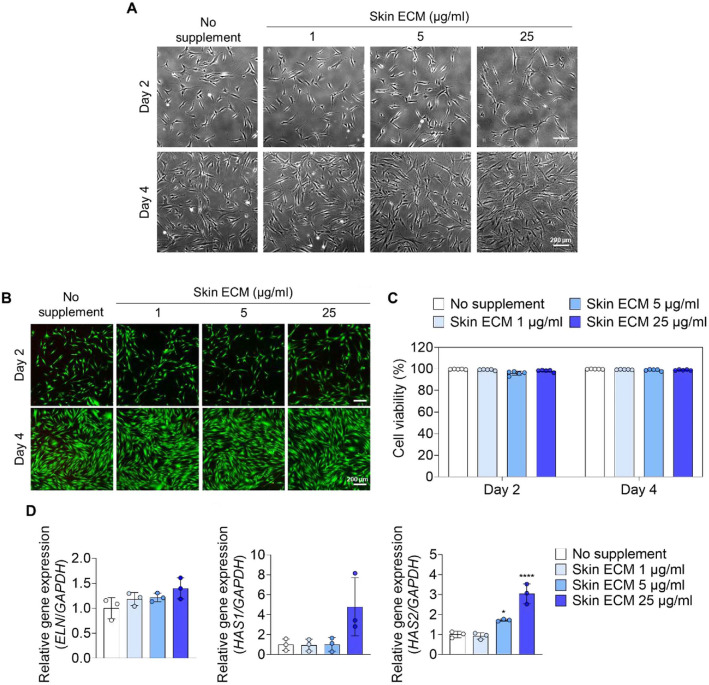
Dose-dependent skin ECM supplementation confirms cytocompatibility and identifies an optimal concentration based on ECM remodeling and hydration gene signatures. (**A**) Brightfield images of human dermal fibroblasts cultured with skin ECM supplementation at varying concentration (0, 1, 5, and 25 μg/ml) on day 2 and day 4. Scale bar = 200 μm. (**B**) Live/dead staining images of fibroblasts following dose-dependent skin ECM supplementation on day 2 and day 4 (Green, live; red, dead). Scale bar = 200 μm. (**C**) Quantification of the live cell fraction based on live/dead fluorescence images (*n* = 5). (**D**) qPCR analyses of fibroblasts treated with dose-dependent skin ECM, showing mRNA expression *ELN*, *HAS1*, and *HAS2* (*n* = 3, *p < 0.05 and ****p < 0.0001 versus no supplement). Data are presented as mean ± SD. Statistical significance was analyzed using one-way ANOVA with Tukey’s multiple comparisons test.

To evaluate cytocompatibility and determine the optimal concentration for skin ECM treatment in human dermal fibroblasts, we first assessed a range of skin ECM concentrations (1-25 μg/ml) administered as a soluble supplement. Fibroblast morphology was well maintained across all concentrations, with no evidence of overt toxicity (Fig. 2A). Cytocompatibility was further supported by live/dead staining, and the skin ECM-treated cells were highly viable at all tested concentrations (Fig. 2B and 2C). Collectively, these data define a safe working range and support skin ECM as a cytocompatible material for skin cells.

Next, to identify the concentration that most effectively promotes a regenerative dermal microenvironment, we quantified the expression of genes associated with dermal ECM remodeling and hydration. Notably, *ELN*, a principal component of elastic fibers that supports skin elasticity, as well as *HAS1* and *HAS2*, key enzymes mediating hyaluronan synthesis that contributes to tissue water retention, showed the highest upregulation following treatment with skin ECM at 25 μg/ml [5, 47-50] (Fig. 2D). Accordingly, 25 μg/ml was selected as the optimal concentration for subsequent experiments.

### Skin ECM Augments Hydration- and Matrix-Remodeling Activities Relative to Skin-Relevant Supplements

To determine whether skin ECM supports skin-relevant cellular responses more effectively than commercial cosmetic ingredients, single growth factors, single-component matrix substrates and cell-derived bioactive materials, we conducted comparative medium supplementation tests in human dermal fibroblast cultures. Two comparator sets were evaluated. The first set comprised FGF-mimetic peptide (ActivePep FG100), EGF-mimetic peptide (ActivePep EF100), RP, PDRN, rhCollagen and VitroCol. The second set included EAA, VEA, HDF exosome, CAcEV, NMN and ADSC-CM. Each comparator was administered as a soluble supplement in parallel with 25 μg/ml skin ECM.

**Fig. 3:**
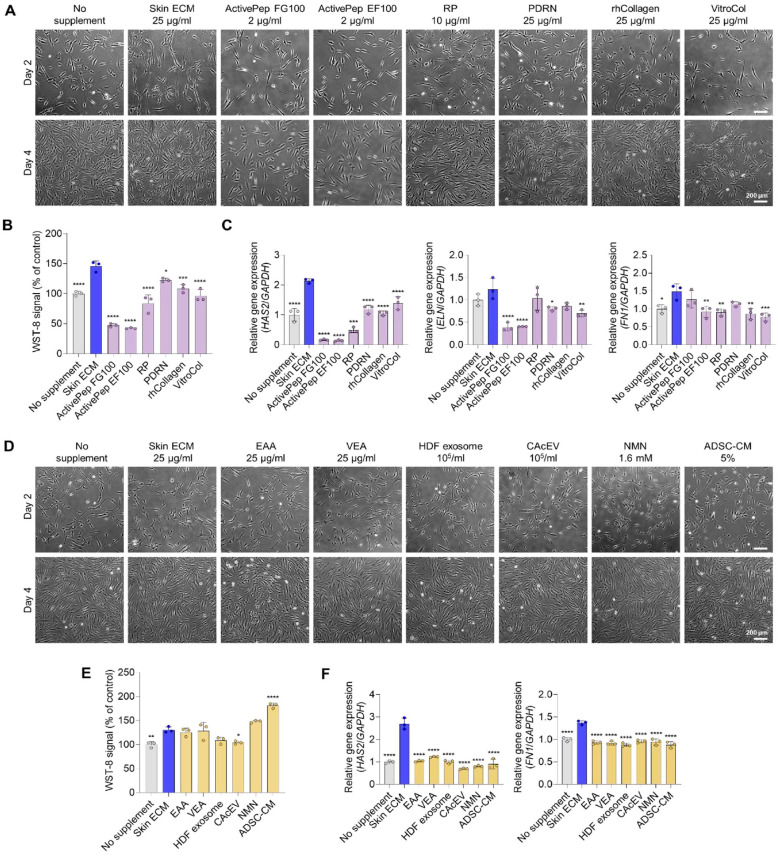
Skin ECM outperforms comparator supplements in inducing matrix-remodeling and hydration-gene programs in fibroblasts. (**A**) Representative brightfield images of human dermal fibroblasts cultured with soluble supplementation of skin ECM (25 μg/ml) and comparator set 1 ‒ ActivePep FG100 (2 μg/ml), ActivePep EF100 (2 μg/ml), retinyl palmitate (RP, 10 μg/ml), polydeoxyribonucleotide (PDRN, 25 μg/ml), rhCollagen (25 μg/ml) and VitroCol (25 μg/ml) on day 2 and day 4. Scale bar = 200 μm. (**B**) WST-8 assay comparing relative metabolic activity at day 4 in comparator set 1 versus skin ECM (concentrations as in (**A**), *n* = 3, *p < 0.05, ***p < 0.001, and ****p < 0.0001 versus skin ECM). (**C**) qPCR analyses of *HAS2*, *ELN*, and *FN1* mRNA expression in fibroblasts treated with comparator set 1 versus skin ECM (concentrations as in (**A**), *n* = 3, *p < 0.05, **p < 0.01, ***p < 0.001, and ****p < 0.0001 versus skin ECM). (**D**) Representative brightfield images of human dermal fibroblasts cultured with soluble supplementation of skin ECM (25 μg/ml) and comparator set 2 ‒ ethyl ascorbic acid (EAA, 25 μg/ml), vitamin E acetate (VEA, 25 μg/ml), human dermal fibroblast-derived exosome (HDF exosome, 1 × 10^5^ particles/ml), *Centella asiatica* callus-derived extracellular vesicle (CAcEV, 1 × 10^5^ particles/ml), nicotinamide mononucleotide (NMN, 1.6 mM) and adipose-derived stem cell conditioned medium (ADSC-CM, 5%) on day 2 and day 4. Scale bar = 200 μm. (**E**) WST-8 assay comparing relative metabolic activity at day 4 in comparator set 2 versus skin ECM (concentrations as in (**D**), *n* = 3, *p < 0.05, **p < 0.01, and ****p < 0.0001 versus skin ECM). (**F**) qPCR analyses of *HAS2* and *FN1* mRNA expression in fibroblasts treated with comparator set 2 versus skin ECM (concentrations as in (**D**), *n* = 3, ****p < 0.0001 versus skin ECM). Data are presented as mean ± SD. Statistical significance was analyzed using one-way ANOVA with Tukey’s multiple comparisons test.

In the first comparator set, fibroblast morphology appeared comparable across all supplementation conditions at both day 2 and day 4 post-seeding (Fig. 3A). However, relative to comparator materials, skin ECM produced the highest water-soluble tetrazolium (WST-8) signal at day 4, indicating enhanced fibroblast viability and metabolic activity (Fig. 3B). This result suggests that skin ECM provides a more supportive microenvironment for fibroblast maintenance than the tested comparators. Moreover, skin ECM treatment induced the highest expression of *HAS2*, a key regulator that contributes to dermal hydration and supports regenerative remodeling (Fig. 3C). *ELN*, associated with skin elasticity and anti-aging effects, and *FN1*, an essential provisional matrix protein involved in wound repair, were also most upregulated in fibroblasts treated with skin ECM compared with other groups [35, 51] (Fig. 3C).

Next, a second comparator set was evaluated using the same experimental workflow. Fibroblast morphology remained similar across all conditions at both day 2 and day 4 post-seeding (Fig. 3D). At day 4, although several comparators resulted in comparable or higher proliferation signals than skin ECM, skin ECM exerted a distinct transcriptional impact compared with the other component conditions. Notably, consistent with the first comparator set, *HAS2* expression was significantly higher in the skin ECM-treated group than in all other conditions, and *FN1* expression was also the highest in the skin ECM group among all supplementation groups (Fig. 3E and 3F). Collectively, these results demonstrate that, relative to the tested skin-relevant comparator supplements, skin ECM elicits a functionally pronounced transcriptional profile associated with hydration and regenerative matrix remodeling, underscoring its potential utility in cosmetic applications improving skin firmness and cutaneous hydration.

### Skin ECM Attenuates Melanogenesis and Reduces Melanin Accumulation

**Fig. 4:**
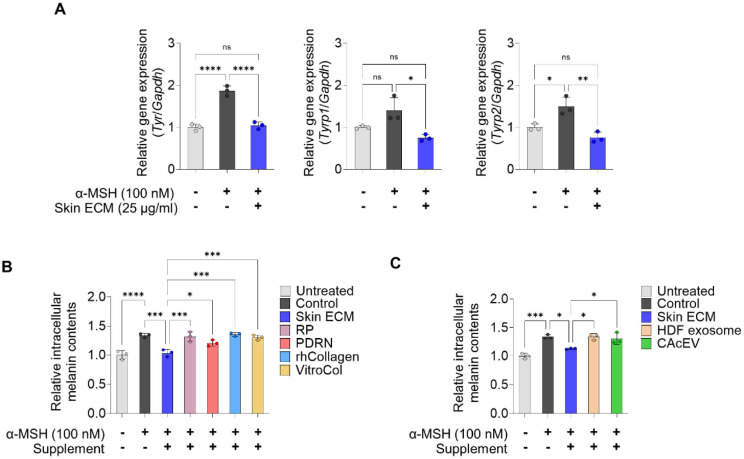
Suppression of α-MSH-induced melanogenic transcripts and melanin accumulation by skin ECM. (**A**) qPCR analyses of *Tyr1*, *Tyrp1*, and *Tyrp2* mRNA levels in B16F10 cells under untreated control, α-MSH stimulation (100 nM), and skin ECM pretreatment (25 μg/ml) followed by α-MSH stimulation, measured at 72 h (*n* = 3, ns: not significant, *p < 0.05, **p < 0.01, and ****p < 0.0001 versus indicated condition). (**B**) Relative intracellular melanin content in B16F10 cells following α-MSH stimulation (100 nM) after pretreatment with skin ECM (25 μg/ml), RP (10 μg/ml), PDRN (25 μg/ml), rhCollagen (25 μg/ml), and VitroCol (25 μg/ml) (*n* = 3, *p < 0.05, ***p < 0.001, and ****p < 0.0001 versus indicated condition). (**C**) Relative intracellular melanin content in B16F10 cells following α-MSH stimulation (100 nM) after pretreatment with skin ECM (25 μg/ml), HDF exosome (1 × 10^5^ particles/ml), and CAcEV (1 × 10^5^ particles/ml) (*n* = 3, *p < 0.05 and ***p < 0.001 versus indicated condition). Data are presented as mean ± SD. Statistical significance was analyzed using one-way ANOVA with Tukey’s multiple comparisons test.

To assess the effects of skin ECM on melanogenesis, murine B16F10 melanoma cells were pre-treated with 25 μg/ml skin ECM for 1 h prior to α-MSH (100 nM) stimulation, and the expression of pigmentation-associated genes was quantified after 72 h. Skin ECM treatment reduced the expression of *Tyr*, a key melanogenic enzyme that catalyzes early steps in melanin biosynthesis, to levels comparable to the control without α-MSH treatment [52, 53] (Fig. 4A). In addition, the mRNA levels of *Tyrp1* and *Tyrp2*, both enzymes involved in eumelanin synthesis and modulation of melanin composition, were also much lower in the skin ECM-treated group than in the only α-MSH-treated group [54]. Together, these findings indicate that skin ECM diminishes α-MSH-induced melanogenic gene expression in B16F10 cells.

Furthermore, to compare the melanogenesis inhibitory effects of skin ECM with those of the comparator supplements, intracellular melanin content was quantified. Following supplementation, skin ECM reduced melanin accumulation in B16F10 cells more effectively than RP-, PDRN-, rhCollagen- and VitroCol-treated groups (Fig. 4B). Skin ECM treatment also resulted in lower melanin levels than HDF exosome- and CAcEV-exposed conditions (Fig. 4C). Overall, these results indicate that skin ECM exhibits a marked anti-melanogenic effect relative to representative skin-relevant comparators, supporting its potential as a skin-brightening cosmetic agent for mitigating hyperpigmentation.

## Discussion

This study supports decellularized skin ECM as a biologically active, skin-mimetic ingredient capable of simultaneously promoting dermal homeostasis in fibroblasts and attenuating melanogenesis in melanoma cells. Notably, relative to other skin-relevant cosmetic ingredients, single-component and cell-derived bioactive materials, skin ECM supported more coordinated cellular responses, reinforcing the premise that dermal tissue-derived ECM supplement can deliver integrated, skin-specific signals for dermal conditioning that are difficult to reproduce with conventional bioactive treatments.

A key distinction revealed by proteomics is that skin ECM retained matrisome diversity that more closely resembles human skin tissue than commercial collagen-based products. While rhCollagen and VitroCol were largely composed of limited set of collagen types, skin ECM preserved a broader spectrum of collagen subtypes together with glycoproteins and proteoglycans involved in matrix regulation, dermal biomechanics, and water retention. This compositional abundance is highly meaningful for cosmetic applications, as skin aging is a multifactorial process characterized by loss of dermal integrity, and homeostasis control [55]. Therefore, supplementation with a single substance may be insufficient to provide complex integrated signals required for maintaining and improving dermal homeostasis, whereas ECM-derived supplement can deliver a richer set of “skin-relevant” cues that collectively shape fibroblast behavior toward matrix maintenance and a more water-retentive states.

Consistent with this proteome-based rationale, skin ECM exhibited excellent cytocompatibility across the tested concentration range in fibroblasts and showed a distinct optimal dose at which dermal function-related genes such as *ELN*, *HAS1*, and *HAS2* were most strongly induced. In supplementation studies, skin ECM further supported high fibroblast metabolic activity and elicited most pronounced upregulation of *FN1*, *ELN*, and *HAS2*, compared with several commonly used cosmetic ingredients. Skin ECM robustly induced hyaluronan-related genes, supporting its potential to enhance hyaluronan-mediated water retention and thereby improve skin moisturization and elasticity/firmness when applied as a cosmetic ingredient. Overall, these results suggest that skin ECM provides skin-mimetic ECM cues that strengthen elastic fiber maintenance and enhance hyaluronan-mediated water retention capacity, consistent with improved dermal quality and hydration-related conditioning.

Moreover, skin ECM also attenuated α-MSH-induced melanogenesis in B16F10 cells, as shown by reduced *Tyr*, *Tyrp1* and *Tyrp2* expression and lower intracellular melanin accumulation relative to several comparators. Although the exact upstream mechanisms were not delineated in this study, the proteomic detection of TGFB1 in skin ECM may provide a plausible compositional link to these anti-melanogenesis outcomes, as TGFB1 signaling has been implicated in hypopigmentation [38, 40].

In summary, our findings support skin ECM as a multifunctional, skin-mimetic ingredient that promotes balanced dermal conditioning across hydration, matrix integrity, and brightening. Compared with other single-component substances and cell-derived bioactives, skin ECM exhibits strong biological activity rooted in the native dermal matrix itself. Because it is largely composed of structural and regulatory components that constitute the cutaneous microenvironment, skin ECM may help sustain a healthier skin milieu by reinforcing intrinsic skin maintenance programs. By supplying these foundational dermal cues in combination, skin ECM may support a steadier healthy-skin state, thereby contributing to improved moisturization, elasticity, and a more even skin tone. Despite these promising findings, a few limitations should be considered. Our study focused on evaluating anti-melanogenic activity in an *in vitro* α-MSH-stimulated B16F10 model; thus, validation in more physiologically relevant systems, including *in vivo* pigmentation models, will be important to more accurately define the anti-melanogenic potential of skin ECM. Accordingly, the observed effects in our study are interpreted as contributing to a more even-looking skin tone rather than indicating direct depigmenting or therapeutic effects. As 2D culture does not fully recapitulate key features of skin tissue, future studies using more skin-mimetic 3D platform, such as organoids, are warranted to better assess efficacy and functional aspects of skin ECM.

## Supplemental Materials

Supplementary data for this paper are available on-line only at http://jmb.or.kr.
